# Predictors of hematoma expansion predictors after intracerebral hemorrhage

**DOI:** 10.18632/oncotarget.19366

**Published:** 2017-07-18

**Authors:** Sheng Chen, Binjie Zhao, Wei Wang, Ligen Shi, Cesar Reis, Jianmin Zhang

**Affiliations:** ^1^ Department of Neurosurgery, Second Affiliated Hospital, School of Medicine, Zhejiang University, Hangzhou, PR China; ^2^ Department of Physiology and Pharmacology, Loma Linda University, Loma Linda, California, USA; ^3^ Department of Preventive Medicine, Loma Linda University, Loma Linda, California, USA

**Keywords:** intracerebral hemorrhage, hematoma expansion, predictor

## Abstract

Despite years of effort, intracerebral hemorrhage (ICH) remains the most devastating form of stroke with more than 40% 30-day mortality worldwide. Hematoma expansion (HE), which occurs in one third of ICH patients, is strongly predictive of worse prognosis and potentially preventable if high-risk patients were identified in the early phase of ICH. In this review, we summarize data from recent studies on HE prediction and classify those potential indicators into four categories: clinical (severity of consciousness disturbance; blood pressure; blood glucose at and after admission); laboratory (hematologic parameters of coagulation, inflammation and microvascular integrity status), radiographic (interval time from ICH onset; baseline volume, shape and density of hematoma; intraventricular hemorrhage; especially the spot sign and modified spot sign) and integrated predictors (9-point or 24-point clinical prediction algorithm and PREDICT A/B). We discuss those predictors’ underlying pathophysiology in HE and present opportunities to develop future therapeutic strategies.

## INTRODUCTION

Intracerebral hemorrhage (ICH), which accounts for 10-15% of stroke, is the most lethal form of stroke with more than 40% 30-day mortality compared to ischemic stroke [1, 2]. Hematoma expansion (HE), which occurs in approximately 33% ICH patients, is identified as one important independent predictor of early neurological deterioration and poor long-term clinical outcomes [3, 4]. However, several therapeutic treatments targeting HE, including early aggressive blood pressure lowering treatment and receiving recombinant activated factor VII (Ravia), have different conclusions of clinical outcome improvement in large randomized controlled trails [5, 6]. For example, in the Second Intensive Blood Pressure Reduction in Acute Cerebral Hemorrhage Trail (INTERACT2), lowering intensive blood pressure showed a borderline significant effect on better primary outcome. Their outcomes may result from the indiscriminate enrollment of patients with low rates of HE. In addition, some patients destined to experience HE were excluded from emergency surgery. Based on that supposition, more attention has been paid on better stratification of patients, especially patients destined to undergo HE. Therefore, it is important to find those HE predictors to stratify patients and tailor intensive therapies timely and effectively for high-risk patient. Several retrospective studies have identified different individual potential predictors using various methodologies, but results are conflicting (Table 1) [7-9]. In this review, we summarize and classify those potential predictors by clinical, laboratory, radiographic, and integrated score models. We discuss their underlying pathophysiology and future therapeutic strategies on HE prediction or prevention.

**Table 1:** Descriptive Summary of Predictors for HE of ICH patients.

Predictors			References	Recruitment Period	Country	Num	Enrollment Window (CT scan)	HE definition	Sensitivity	Specificity	PPV	NPV	OR(95%CI)	AUC
Maximum SBP		Ohwaki,2014[72]	1998-2002	Japan	76	second <48h	>40% or >12.5 mL	/	/	/	/	1.04(1.01-1.07)	/
CRP>10mg/L		Di Napo,2014l[100]	2009-2011	International	399	first <6h	>33% or >12.5 mL	/	/	/	/	4.71(2.75-8.06)	/
C-Fn>6µg/mL		Silva,2005[42]	NA	International	183	symptom onset <12h	>33% for <20mL; >10% for ≥20mL;	/	/	/	/	92(22-381)	/
IL-6>24 pg/mL								/	/	/	/	16(2.3-119)	/
Density in CT		Barras,2009[114]	NA	International	90	first <3h	>33% or >12.5 mL	65.6%	46.6%	40.4%	71.1%	/	/
Shape in CT								78.1%	20.1%	35.2%	63.2%	/	/
mNIHSS		Chan,2015[63]	2008-2010	USA	257	first <24h, second <48h	>33% or >12.5 mL	/	/	/	/	1.06	0·6712
Warfarin use								/	/	/	/	1.9	0·6712
Warfarin use		Yaghi,2014[12]	2009-2012	USA	200	first <12h, second <24h	>33%	/	/	/	/	3.6(1.3-10.3)	/
IVH								/	/	/	/	5.7(1.5-20.9)	/
Spot sign		Orito,2016[134]	2012-2013	Japan	80	NA	>10%	77.8%	73.8%	83.3%	46.8%	/	/
Spot sign		Andrew,2012[17]	2006-2010	6 countries	268	first <6h	>33% or >6 mL	51%	85%	61%	78%	/	/
High HU of spot		Kim,2014[143]	2009-2011	Korea	316	NA	>33% or >6 mL	/	/	/	/	1.048(1.01-1.09)	/
Spot sign number	≥1	Huynh, 2013[142]	2006-2010	6 countries	268	first <6h	>33% or >6 mL	51%	85%	61%	78%	/	/
	≥2							32%	92%	64%	74%		
	≥3							12%	97%	64%	70%		
	≥4							3%	99%	50%	68%		
Short initial time		Kim,2014[143]	2009-2011	Korea	316	NA	>33% or >6 mL	/	/	/	/	0.197(0.06-0.61)	/
Time from onset to CT	0-2h	Dowlatshahi,2016[109]	1946-2016	International	1039	NA	>33% or >6 mL	60%	76%	61%	76%	/	0.68
	2-4h							55%	84%	57%	82%		0.69
	4-6h							44%	91%	56%	87%		0.68
	6-8h							56%	92%	64%	90%		0.74
	>8h							30%	90%	33%	89%		0.60
CTP spot sign		Koculym,2013[136]	Six months	Canada	28	first <6h	>30% or >6 mL	78%	100%	100%	71%	/	/
Leakage sign		Orito,2016[134]	2012-2013	Japan	80	NA	>10%	93.3%	88.9%	94.3%	66.7%	/	/
Spot & Leakage sign								93.8%	91.4%	97.1%	68.9%	/	/
Black hole sign		Qi,2016[140]	2011-2015	China	206	first <6h, second <30h	>33% or >12.5 mL	31.9%	94.1%	73.3%	73.2%	/	/
Score Models	9-point	Huynh,2015[83]	2006-2012	6 countries	301	first <6h	>33% or >6 mL	/	/	/	/	/	0.761
	24-point												0.673
	PREDICT A												0.823
	PREDICT B												0.804

HE indicates hematoma expansion; PPV, positive predictive value; NPV, negative predictive value; SBP, systolic blood pressure; CT, computed tomography; CRP, C-Reactive Protein; C-Fn, cellular fibronectin;IL-6, interleukin-6;NA,not available; mNIHSS, modified National Institutes of Health Stroke Scale; IVH, intraventricular hemorrhage; HU, Hounsfield Unit; CTP, CT perfusion.

## THE DEFINITION AND PATHO-PHYSIOLOGY OF HEMATOMA EXPANSION

The definition of HE varied in different literatures with a 40% relative volume increase or 12.6mL absolute volume increase in hematoma size from baseline CT to follow-up CT in one [10], a 33% relative increase in others [11, 12], or even a 50% relative increase and 2mL absolute increase in another [13]. One study found absolute HE definitions have higher positive predictive value for poor outcome than relative definitions. This could be explained because absolute HE is directly proportional to the truly damaged brain tissue [14]. Furthermore, they found that baseline hematoma size doesn’t influence the prediction of absolute and relative HE definition. This overturns the expectation that the absolute volume increase would be greater in large baseline size hemorrhages and absolute definition of HE would predict better in small baseline sizes. So using a method that assigns equal weight between absolute and relative definition could maximize sensitivity and specificity [15]. The INTERACT1 study categorized absolute and relative HE from minimal change (<5mL or <33%) to massive change (>12.5mL or >50%) [2]. Some studies on CT angiography contrast extravasation defined HE as a proportional increase of 33% or an absolute increase of more than 6mL [16-18]. One study mathematically derived “optimal” HE cutoff was ≥ 3mL for absolute growth (sensitivity 49%, specificity 81%) and ≥ 26% for relative growth (sensitivity 42%, specificity 80%) [14]. Interestingly, these “optimal” cut points may not be the most clinically suitable for use because a clinical relevant definition of HE should have a high positive predictive value, not equal weight of sensitivity and specificity. Thus, they assume the best clinical definition of HE was its dichotomized definition: relative (>33%) or absolute change (>12.5 mL) comparing with hematoma volume from initial to following CT slide, and applied in the large clinical trial like INTERACT2 and rFVIIa ICH trail [19, 20]. The relative cutoff was chosen prospectively for three reasons: First, a 10% change in diameter corresponds to a 33% growth in the sphere volume, which makes a clear difference to the naked eye of a physician viewing serial CT scans. Second, different positions and angles of the CT slice images could cause up to one-third “decrease” volume effect between the baseline and 1-hour CTs, especially for small hemorrhages. So the relative CT definition would represent the true HE, not volume variability of small hematoma in CT imaging [21, 22]. Third, this cutoff has been applied in the large clinical ATACH-I and II trials consistently [11]. The absolute cutoff was ≥12.5mL. Though absolute ≥ 12.5mL change was only seen in less 20% of patients, it’s a necessary trade-off having fewer patients for better positive predictive value, odds ratio, and detection. Despite the relative (>33%) or absolute change (>12.5 mL) combination definition was most used in recent studies, the preferred cutoff for clinically significant HE has not reached a consensus. Thus we believe mathematically or clinically optimal cutoffs could be validated, not only to lower the heterogeneity among studies, but also to better stratify high-risk patients.

**Figure 1:**
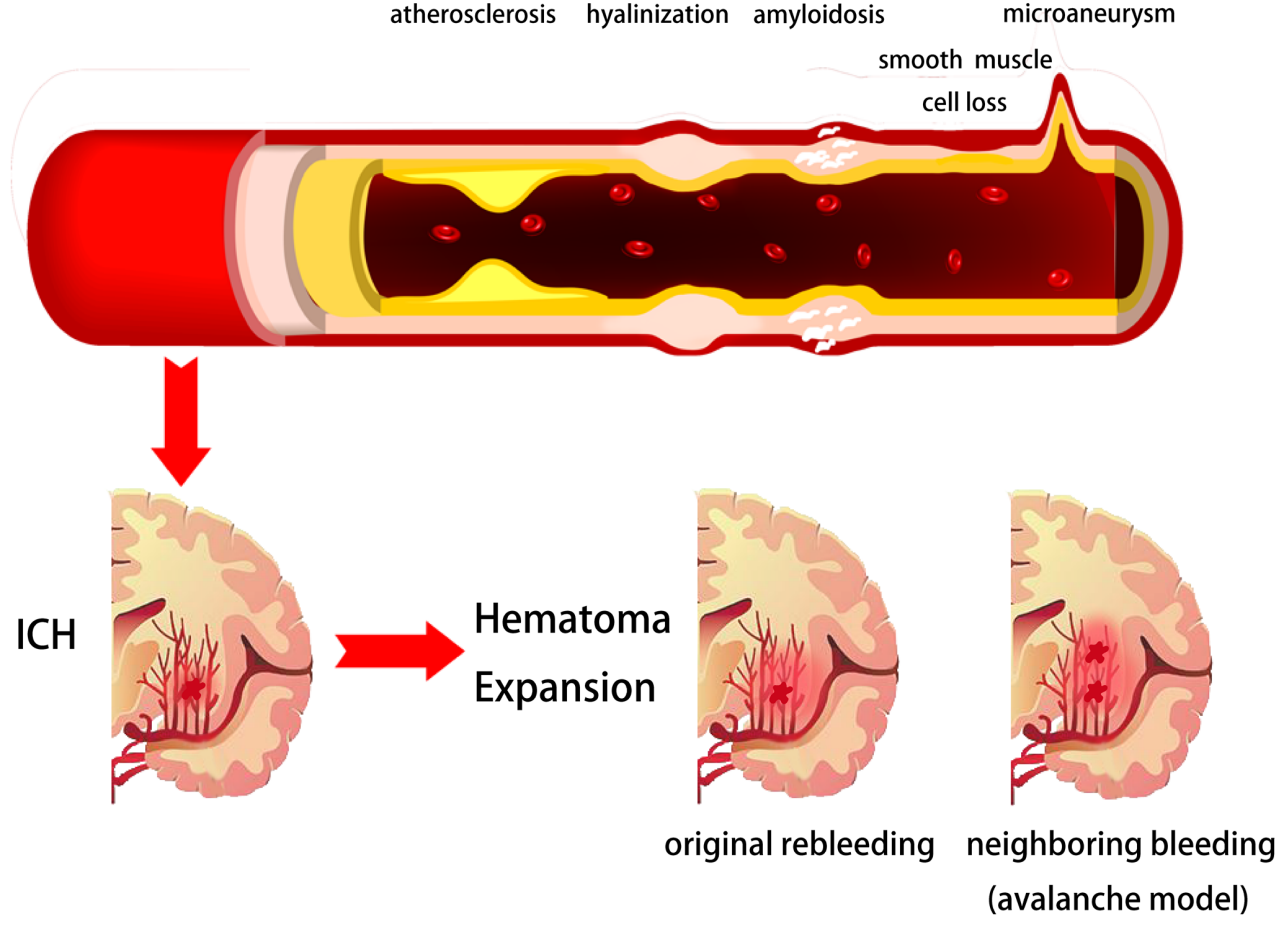
Potential pathophysiological mechanisms of intracerebral hemorrhage and hematoma expansion Arterial pathophysiological changes like atherosclerosis, hyalinization, amyloidosis, smooth muscle cell loss and micro-aneurysm could be the underlying reasons of intracerebral hemorrhage. The original site ongoing bleeding or perihematomal vessels bleeding may be the primary force of hematoma expansion.

HE presents the extended distribution of initial hemorrhage, including ventricle invasion, transition or rebleeding into compartments adjacent to the initial zone and intraparenchymal volume growth. HE occurs in about 73% of patients within 3 hours from symptom onset and clinically prominent expansion occurs in 35% of patients [13, 22, 23]. However, the pathophysiology of HE remains unclear and needs further investigation. Previous studies have proposed several mechanisms of HE, including ongoing bleeding, coagulopathy state within hematoma, as well as rupture of perihematomal vessels [24-27]. Leakage or rebleeding from one or more ruptured arteries may be the primary force of HE. In 1971, a study using microscopic serial sections on pontine and putamen hemorrhages have proposed an “avalanche” model for HE [28]. This model describes the HE as secondary mechanical shearing of periphery vessels caused by expansion of initial bleeding. Clinical observation, genetic data, radiologic evidence, and computational simulation supports this model [29-32]. Within the first hour, hematomas induce brain injury by neuronal and glial mechanical disruption, oligemia or ischemia, which induce neurotransmitters to release. In addition, mitochondrial failure, through glutamate release and calcium influx, resulted in sodium accumulation, cytotoxic edema, and necrosis within the first 4 hours [1, 33, 34]. Products of hemoglobin breakdown and coagulation, such as but not limited to thrombin, ferrous, iron, and heme initiate a secondary injury cascade [1, 33, 35]. In particular, thrombin activated microglia to release oxygen free radicals (OFR), tumor necrosis factor α (TNFα), interleukin-1β (IL-1β), matrix metalloproteinase (MMP), and complement factors to trigger blood-brain barrier (BBB) connective tissue breakdown, astrocytes aquaporin-4 (AQ-4) expression, and neuronal and glia apoptosis [36-39]. Those physiological processes induce vasogenic edema, polymorphonuclear neutrophil (PMN) and macrophage recruitment [1, 36, 40, 41]. Those ICH-initiated injury cascades: secondary inflammation elicited by degradation products and alterations in BBB basal membrane by MMP induction, are suggested to cause hematoma enlargement [36, 42, 43]. Furthermore, coagulopathies, oral anticoagulants, autoregulation failure, and uncontrolled perfusion pressure might account for HE by repeated or continuous bleeding. Since these mechanisms are all pathologically plausible, further investigation focusing on the precise mechanism of HE is required [44, 45] (Figure 1).

## CLINICAL FEATURES

Previous studies have demonstrated several clinical features related with HE, such as consciousness level, blood glucose, and blood pressure.

### Consciousness level

Glasgow Coma Scale (GCS) and National Institutes of Health Stroke Scale scores (NIHSS) are identified as predictors of mortality and severe disability [13, 46, 47]. GCS and NIHSS, the routinely estimated clinical data, are also identified as HE predictors in some studies [13, 23, 46] although this relationship is infrequently reported in large trails [16, 48, 49]. We notice that consciousness disturbance is more likely normal course after ICH symptom onset and exacerbates along with HE. In addition, lower CGS and greater NIHSS could represent a number of factors, especially the larger initial ICH volume, a recognized predictor of HE [16, 49]. Furthermore, clinically emergent craniotomy and hematoma evacuation were performed for patients with poor neurological condition, like GCS score < 8 and not performed for patients with poorest consciousness level, like GCS score of three with bilateral dilated pupils. Despite remaining controversy in the relationship of HE and consciousness level, low consciousness level could already help surgeons select patients for more intensive strategies, regardless of whether or not HE occurs.

### Blood glucose

More than 50% of patients with stroke have hyperglycemia [50]. Several studies have indicated relationship between high blood glucose at admission with high mortality, regardless of whether or not they are diabetic [51-53]; However, the relationship between elevated glucose and HE in ICH remains controversial. Some analysis found a univariate association between lowering serum glucose concentration and decreased HE, as well as perihematomal edema and outcome [54, 55]. Hyperglycemia increases the magnitude of HE in experimental ICH model [56, 57]. For the pathology of hyperglycemia, transient hyperglycemia following stroke is hypothesized as a transient stress reaction to ICH processes, inflammation, or some other mechanisms [58, 59]. Some studies show the deleterious effects of hyperglycemia are attributed to its secondary promotive effects of acidosis, free radical formation, and inflammatory cytokines release. Those secondary effects accelerated the BBB breakdown, impaired the integrity of adjacent vessels surrounding the initial bleeding site, and promoted emerging or continuous bleeding [53, 54, 56, 57, 60-62]. Those physiological processes are interrelated with HE as well and will be discussed below respectively. While other studies show different results [12, 63]. One study shows patients with or without HE have similar blood glucose in mg/dL (179±68 vs 153±71) and diabetes (14% vs 25%) [12]. Thus, the intrinsic mechanism of high serum glucose and HE needs to be explored in future studies. Whether hyperglycemia is the response or the cause of hematoma growth needs to be elucidated. Several studies have shown that not only hyperglycemia at admission, but hypoglycemia also occurred in intensive glucose control and could increase mortality among critically ill patients [64-67]. Thus, both clinical hypoglycemia and hyperglycemia should be avoided. The optimal management of hyperglycemia in ICH and the target glucose level call for extensive basic studies and large scale clinical trials [68]. The major problem to be revolved in future is the internal pathogenies between blood glucose and HE.

### Blood pressure

Elevated blood pressure at admission of ICH patients is positively associated with poor outcomes, due to its automatic pressure to the arteries and elevation effect on intracranial pressure [69, 70]. Some studies found that hypertension is more likely to be associated with large hematoma volume and HE, which make early blood pressure a potential target for better outcomes by mitigating HE [6, 7, 71-74]. One genetic research study demonstrated that increasing numbers of high blood pressure-related alleles are associated with mean baseline hematoma volume and poor clinical outcome in ICH [75]. But unfortunately, several studies and large clinical trials INTERACT 1/2 failed to reach conventional statistical significance between lowering blood pressure earlier and improving primary outcome [5, 76-78]. Further analysis of INTERACT1 indicated that early treatment to lower blood pressure was able to attenuate HE at a 1.7 and a 3.4 mL absolute volume in HE within 6 and 4 hours respectively, which could be expected to result in at least 8% and 15% relative increased chances of better outcome. The main-phase study, INTERACT2 showed that the difference in HE between intensive-treatment group (with a target SBP <140 mmHg within 1 hour) and stand-treatment group (with a target SBP <180 mmHg) was not significant after adjusting the prognostic factors. INTERACT2 failed its primary endpoint of fatality and disability rate reduction, but in the key secondary endpoint of the distribution of Modified Rankin Scale (MRS), there was a significant favorable shift in intensive pressure control group [5]. The 5-year, multi-center, 1000 patients enrolled ATACH II clinical trial is expected to provide essential information regarding the efficacy of early intensive antihypertensive treatment using intravenous nicardipine. And the underlying mechanism for its expected beneficial effect is presumably mediated through reduction in the rate and magnitude of HE in acute ICH patients [11]. Recently, the ATACH II have reported that the primary outcome of death or disability was 38.7% in the intensive pressure reduction group (target of 110 to 139 mmHg) and 37.7% in the standard pressure reduction group (target of 140 to 179mmHg). In addition, the percentages of patients with HE didn’t differ significantly between two groups (18.9% in intensive group, 24.4% in standard group, adjusted risk ratio, 0.78;95%CI, 0.58-1.03) [79]. As for the clinical practice, early aggressive acute BP lowering is proven to be safe, with no more chance of exacerbation of cerebral ischemia, infarction, or perihematoma hypoperfusion [80-82]. In summary, the beneficial effect of early blood pressure control for outcome through HE prevention needs further elucidation.

## LABORATORY PARAMETERS

Laboratory parameters, which focus mainly on the aspects of coagulation status, inflammation, and microvascular integrity, draw more and more attention to reveal their relationships with HE based on the consideration of their mechanism rationality, detection applicability and simplicity.

### Coagulation status

Altered coagulation status may increase the risk of surrounding vessel bleeding after injury and this influence will persist beyond 24 hours, which could result in volume growth at present and future observation [79]. The low level of fibrinogen, the high level of D-dimer and international normalized ratio (INR) >1.5 seem to be predictors of HE [13, 83]. Fibrinogen plays an important role in both primary (aggregate Glycoprotein IIa/IIIb coupled-platelet) and secondary hemostasis (cross-link each other to form fibrin polymers). D-dimer, as a marker of fibrin turnover, reflects disturbance in the coagulation and fibrinolysis pathways. Higher INR means longer prothrombin time, which means worse coagulation function and more time for plasma to clot after tissue factor addition.

Several retrospective studies demonstrated a negative relationship of prior antiplatelet use with HE and clinical outcomes [8, 49, 84, 85]. One study reported that aspirin use was associated with HE and mortality, but not with functional outcome at 3 months [85]. One retrospective analysis of 251 patients demonstrated that antiplatelet therapy was an independent predictor for the occurrence of hematoma enlargement, emergent death, and evacuation surgery [86]. While in the Cerebral Hemorrhage and NXY-059 Treatment (CHANT) trial, antiplatelet medication is not associated with increased hemorrhage volume, HE, or clinical outcome at 3 months [87]. Studies demonstrate that vitamin K antagonists are related to higher risk for HE and poorer clinical outcome [8, 49, 84, 85]. The measurements of anti-warfarin effect on HE, such as Prothrombin Complex Concentrates (PCC), Fresh Frozen Plasma (FFP) and targeted therapies (such as rFVIIa) are brought to the forefront. Some studies show PCC use in ICH patients could have faster INR reversal and less HE occurrence than FFP or vitamin K [88, 89] because FFP and vitamin K take nearly 24 hours to normalize INR [90-92]. Conversely, PCC could reverse INR within minutes, and rFVIIa may be faster than PCC [91-94]. The failure to reverse the INR within 2h is an independent predictor of death or severe disability, and rFVIIa could reverse INR within minutes [95]. Based on those considerations, rFVIIa was recommended as a potential ultra-early hemostatic intervention before coagulopathy and auto-regulation failure occurs. The rFVIIa ICH trail demonstrated early administration of rFVIIa (<4h of symptom onset) has a substantial reduction of HE at 24h (3.3mL, 4.5mL, and 5.8mL in the group 40μg, 80μg and 160μg rFVIIa per kilogram, respectively comparison with placebo group), better outcome at 3 months, and decreased mortality rate by 38% [6]. Based on those facts, we believe impaired coagulopathy increase the risk of HE and earlier recovery of it by faster INR normalization may represent less HE and better outcome. Theose safe and effective clinical interventions based on coagulopathy targets are the future directions.

### Inflammation and microvascular integrity

Increasing evidence has shown that inflammatory responses and microvascular integrity damage participate in the pathophysiological processes of brain injury following ICH [96]. Multifocal bleeding around the circumstances of clot has induced hematoma growth [24]. Those clots, caused by the rupture of arterioles and venules, induce the thrombin generation. In experimental ICH model, thrombin activates the inflammatory cascade and the expression of matrix metalloproteinase (MMPs) [97, 98]. MMPs could cause the basal membrane components degradation, BBB damage, and brain edema. Inflammatory markers on admission, such as elevated white blood cell count, interleukin-6 (IL-6), C-reactive protein (CRP) are found to be associated with worse outcomes and HE [42, 99, 100]. One study has shown that patients with HE have significantly higher plasma concentrations of IL-6, TNF-α, MMP-9, and cellular fibronectin (c-Fn) than patients without HE [101]. c-Fn, as a glycoprotein, is one of basal membrane components, which is important for platelets’ adhesion to fibrin and blocking further bleeding. Notably, the IL-6 level (> 24 pg/mL) and c-Fn level (>6µg/mL) in the peripheral blood increased 16-fold and 92-fold the risk of HE, respectively [42]. Even more, IL-6 is the major inducer of CRP. Another study found CRP (>10mg/L) causes higher risk of HE (adjusted risk ratio, 4.7;95%CI, 2.8-8.1) than the spot sign (adjusted OR, 2.3;95%CI, 1.6-3.1) [100]. From the physiological standpoint, the acute reactant CRP can disrupt BBB to promote brain edema and HE formation. On one hand, the disappearance of basal membrane components and loss of microvascular integrity could aggravate inflammatory reaction [102-104]. On the other hand, it’s likely that even a mildly inflammatory status could damage coagulation function and vessel wall pathophysiology as well [99, 105]. Despite the underlying mechanisms aren’t fully understood so far, we believe inflammation and microvascular pathophysiology have mutual promotion effects and both can contribute to vessels’ persistent leakage, the formation of brain edema and HE subsequently. Based on the fact that those molecular signatures of vascular injury and inflammatory response are predictive of subsequent HE and easily measured in the first hours after symptom onset, we believe those molecular predictors may open new potential hemostatic and anti-inflammatory therapeutic strategies for ICH despite many underlying mechanisms still require future investigation.

## NEURORADIOLOGICAL CRITERIA

The urgent, rapid-developing abnormalities and unconsciousness could suggest the diagnosis of ICH. But the imagings of the brain, particularly CT scan, are vital to diagnose ICH and distinguish ICH from cerebral infarction. The initial CT scan at admission could inform emergency surgeons the location and size of the hematoma, the presence of intraventricular hemorrhage and the occurrence of hydrocephalus. Conventional angiography is recommended to look for secondary causes of ICH, such as aneurysms and arteriovenous malformation. The follow-up CT, normally acquired within 6-72 h after the first CT, is usually used for ICH prognostication and HE diagnosis. In short, imaging characteristics on CT scan remain the hot research spot in the last decade.

### The interval time from ICH onset

Computed tomographic angiography (CTA) and gadolinium-enhanced magnetic resonance imaging (enhanced MRI) studies confirm 46% and 36% of patients had active contrast extravasation within the hematoma, respectively. Those patients had a significantly less time interval between symptom onset and higher mortality (64% versus 16%) [106-108]. Some studies noticed that active bleeding seems to be stabilized within 6h and the incidence of HE decreases as the interval time from onset increases. It means that the interval time from ICH onset to admission or first CT scan is a strong predictor of HE [16, 109]. One prospective cohort study of 1000 patients proved that shorter time to initial CT (≤6 hours) means greater possibility of HE than later presentation (>6 hours) (adjusted odds ratio, 2.55;95%CI, 1.53-4.27) [16]. One explanation is that ICH is a dynamic illness. Those patients with HE may be still in the acute period of ICH and the bleeding is still ongoing. The contrast-enhanced imaging could indicate the active bleeding process in ICH. Initial CT scan could be applied before the hematoma formation and expansion stabilized, in order for those patients to be treated timely in their acute course of the fatal disease. In short, the earlier the first scan did, the greater the probability of later HE became. We think future research could focus on whether different interval time from onset to first scan and first scan to second scan influence HE definition and other factor’s predictive abilities.

### The baseline ICH volume

ICH volume has significantly positive correlation with short term and long term mortality [47, 110-112]. Although the criteria for hematoma volume for favorable prognosis is still unclear, many studies demonstrated large volume (>30mL) is associated with poor outcomes at discharge and at 1 month follow-up [1, 47], while small volume (<30mL) means favorable outcomes [47, 113, 114]. Researchers propose that the internal connection may be the elevated intracranial pressure and cerebral edema, which are positively related to greater initial ICH volume. Unfortunately, despite perihematomal edema volume and baseline hematoma volume being intimately related, perihematomal edema volume has conflicting results on its independent predictive ability of clinical outcomes and HE [115-118]. One alternative assumption is that early HE, not perihematomal edema volume, may be the association between greater baseline ICH volume and higher morbidity and mortality. Large ICH volume (>30mL) is more likely to associate with HE, and HE is less likely in small hematoma (<10mL) [14]. From the multivariable analysis of the prediction score for HE, baseline ICH volume 30-60mL and >60mL have more adjusted odds ratio than ICH volume <30mL (1.64;95% CI, 1.04-2.59 and 2.10;95% CI, 1.25-3.55) [16]. A larger baseline ICH volume may indicate multiple bleeding artery points high systemic arterial pressure and further induction of perilesional hemorrhage through additional vessel shearing and the avalanche effect of HE [119]. Genetic studies have proven apolipoprotein E (APOE) ε2 allele one allele of the principal cholesterol carrier in the brain can promote vasculopathy in cerebral amyloid angiopathy and HE in ICH [120, 121]. A reasonable speculation is that this allele may increase the vascular walls’ fragility to the secondary neighboring vessel mechanical shearing effect, just like the strong correlation between this allele and an elevated disruption of vessel walls promoted by enhanced by cerebral amyloid angiopathy [122-125]. The increasing baseline ICH volume on initial CT scan is associated with higher likelihood of increased absolute ICH volume growth on the secondary scan [16]. We think those open problems could be solved in future studies: better ICH volume measurements, effective cutoff of large-small volume and internal mechanism of large volume.

### The shape and density of hematoma on CT

Irregular shape and density of hematoma on CT scan seem to predict HE [13, 114]. Regular lesion edge indicates a solitary focus hematoma and irregular shape bespeaks the hematoma arises from multiple foci. In addition, heterogeneous density on CT also indicate active hemorrhage or multiple bleeding vessels, which is the underlying mechanism of HE. In one study, using 2 novel 5-point categorical scales reflecting the spectrum of appearance of ICH shape and Hounsfield Unit (HU) density variation, they found median growth(10-25mL) was higher in the heterogeneous group than the homogeneous group. And the irregular group had higher median growth than regular group. Furthermore, adjusting for the two independent predictors (baseline ICH volume and time to scan), density heterogeneity, not shape irregularity could predict HE on the continuous growth scale [114]. Considering the qualitative description of the shape and density on CT will vary greatly under different scan times, ICH initial volume, and observer diagnosis bias, not only time course of ICH and HE definition need to be elucidated, but the standardize morphology scale should be established for its feasibility and convenience for clinical practice [13, 22].

### Intraventricular hemorrhage (IVH)

The risk of IVH becomes particular high when ICH has been noticed in close proximity to ventricular system, like thalamic hemorrhages [126]. Previous studies have found that the presence of IVH over the first few days after the symptom onset is associated with elevated mortality and worse clinical outcome [127-129].

One study demonstrates that patients with HE are more likely to have IVH (79% vs 45%) by univariate analysis. IVH is significantly predictive of HE (OR=5.7,95%CI:1.5-20.9) [12]. One hypothesis conveys that IVH tends to trigger pro-inflammatory cytokine activation combined with systematic and local homeostatic/fibrinolytic pathways alternation [25, 130, 131]. ICH patients with IVH have elevated levels of white blood cell count, thrombin-antithrombin complex, plasmin-antiplasmin complex and D-dimer, which collectively indicate the infection and stress states of the coagulation/fibrinolytic system [130]. The potential ultra-early hemostatic intervention, rFVIIa, can reduce the risk of HE in IVH (17% and 10% of placebo and rFVIIa treated patients) [126]. So far, the mechanism of systemic activation of hemostatic and inflammation systems in IVH patients and clinical measures to prevent HE based on this hypothesis need further studies to elucidate.

### The spot sign and modified spot sign

The phenomenon of ICH, spot sign, has been studied extensively in the last decade and might be the strongest individual predictor for subsequent growth of hematoma. Although spot sign has diverse definitions, it’s mostly denominated as at least 1 tiny focus of contrast pooling within the ICH and high Hounsfield Unit value (>120), discontinuous from normal or abnormal vasculature adjacent to the ICH and any size and morphology on CTA scan in most studies [132-134]. It has been validated that spot sign is a crucial imaging biomarker for HE and poor clinical outcome. Although the definition of spot sign and HE, even the timing of image acquisition, are not used constantly across literatures, CTA “spot sign” still had 51%-98% sensitivity and 50%-89% specificity in previous studies [9, 18, 135]. Further studies describe the spot sign as a dynamic entity. One plausible explanation is that spot sign might represent active bleeding or rebleeding through ruptured vessels, which reflects contrast extravasation on CT or MRI scan. Based on this mechanism, many modified spot signs were introduced, such as delayed CTA, venous phase CTA, dynamic CTA, post contrast CT (PCT), and CT perfusion (CTP) [135, 136]. Higher sensitivity and specificity was obtained by the combination of initial and modified CTA, regardless of the heterogeneity of secondary CT time and definition of spot sign. For example, CTP spot sign was proven as an independent predictor of HE and poor outcome (OR,13.7;95%CI,1.12-166) and higher sensitivity (78%) compared with CTA spot sign (44%) or PCT spot sign (50%). CTP’s improved sensitivity was mainly attributed to its moderate delay time (30-70 seconds for CTP, 20-26 seconds for CTA, and 300-360 seconds for PCT), which is enough for contrast to extravasate and not too long to diffuse or get subthreshold [136, 137]. Considering MRI can accurately detect acute and chronic ICH, one study investigated that spot sign detected on post-contrast T1-weighted and dynamic T1-weighted scan is also a potent predictor of the clinical outcome. But no significant HE differences were observed between spot sign and non-spot sign group (52.2% in spot sign group, 40.7% in non-spot sign group) [138]. Further research about MRI spot sign is needed due to this study’s small sample size, lack of long-term follow-up, and observational study design.

Based on the relatively lower sensitivity of spot sign and better results in modified spot signs (2 to 5 minutes delayed-phase CT) [31, 139], one study presents the leakage sign, which is defined as a 10% increase in HU of a 1-cm diameter restrictive area between CTA initial phase and delayed phase (specifically 5minutes after the initial phase). They found leakage sign has higher sensitivity (93.3%) and specificity (88.9%) for HE than the spot sign (77.8% sensitivity and 71.8% specificity) [134]. A study enrolling total 206 patients found that the black hole sign, 28 HU difference between the relatively hypo-attenuated area and adjacent hyperintense area on CT scan, is found more commonly in HE patients (31.9%) than those without HE (5.8%) [140]. The sensitivity and specificity value of black hole sign’ HE prediction are 31.9% and 94.1%, respectively. The black hole sign, a simple and easy-to-use CT predictor, has improved reliability and subjectivity compared with previous spot sign description and this improvement can be explained by the plausible pathophysiological process (hypo-attenuated fresh liquid blood turning to hyperintense “old blood” with clot retraction and serum dissociation) [27]. The optimal HU thresholds for leakage sign and black sign need further identification through more studies. More importantly, the spot sign score (SSS) is devised from three most prognostic radiological characteristics for HE: spot number (1-2 for 1 point, ≥3 for 2 points), maximum spot size (axial dimension 1-4mm for 0 point, ≥5mm for 1 point), and density heterogeneity. SSS reports an accurate risk stratification of hematoma expansion as well as a reliable independent prediction of mortality and poor clinical outcome [31, 141, 142]. Further multicenter external validation of the SSS from analysis of a multicenter prospective observational cohort study, the PREDICT study, demonstrates that spot sign number is the most predictive spot characteristic among the three radiological characteristics (p<0.001 for number, p=0.570 for maximum density and p=0.982 for maximum axial dimension by comparison HE group with non-HE group) [142]. Increased spot number shows significant positive association with absolute HE and relative HE (both p<0.001), whereas SSS category demonstrates no significant trends for absolute HE and relative HE (p=0.182 and 0.470, respectively). In conclusion, those useful radiological signs, such as spot sign, modified spot sign, leakage sign on CTA, black hole sign on CT, spot sign number are all the crucial predictors for HE and prognosis. And further exploration of other radiographic characteristics may establish a more reliable, objective, and convenient predictor.

## PREDICTION SCORE MODEL

Some prediction scores have recently been developed and validated for HE prediction. A 9-point clinical prediction score is established on four recognized predictors: time to presentation, anticoagulation use, ICH volume, and CT angiography spot sign, all of which could be explained by the theoretical HE model described in 1971 [28]. 80% of patients with highest score of 9 have HE, while the incidence of HE reaches only 5.7% for patients with lowest number 0. The high score (4-9 vs 0-3 in the 9-point score) has strongly positive relation with greater risk of HE (OR, 4.59; 95%CI, 2.06-10.22) [16]. In-hospital and 3-month mortality are increased steadily with the higher prediction scores. This prediction score extends the enrollment time window for trials (>6 hours) and allows more patients to receive therapeutic benefit, without restricting intervention to the first hours after symptom onset. Similarly, a 24-point clinical prediction algorithm (BRAIN) is derived and validated from sub-studies of INTERACT1 and 2 [49]. This BRAIN score predicts the probability of HE (>6mL) within 24 hours after symptom onset, using multivariable logistic regression to identify 5 routinely assessed clinical factors: baseline ICH volume, recurrent ICH, anticoagulation with warfarin at symptom onset, IVH and time to have first-CT scan (only included patients within 6 hours), to predict the possibility of HE (3.4% for 0 point and 85.8% for 24 points). Of note, despite the most robust predictor of HE to date, spot sign on baseline CTA is not included, the 24-point prediction score still shows good discrimination and calibration (C-statistic, 0.73), comparable with 9-point score (0.72 for the development cohort and 0.77 for the validation cohort). Because GCS and NIHSS were often collected clinically and were also identified as HE predictors [13, 46], they were incorporated into two new HE prediction scores, PREDICT A and B, respectively [83]. Both prediction scores still included GCS or NIHSS, warfarin use or INR>1.5, spot sign number, and time from symptom onset to baseline CT. Compared with the 9-and 24-point scores, both PREDICT scores showed improved AUC for HE> 6mL or 33% discrimination compared with the 9- and 24-point scores (PREDICT A 0.78; 95%CI,0.73-0.84; PREDICT B 0.77; 95%CI,0.72-0.83; 9-point score 0.71; 95%CI,0.65-0.77; 24-point score0.76; 95%CI 0.70-0.82;). The delayed contrast enhanced image, like dynamic CTP and post-contrast CT, are still excluded in those prediction scores despite their improved sensitivity and specificity for HE prediction. To stratify patients accurately for current therapeutic intervention, further independent validation and modification of the aforementioned prediction scoring systems is required.

## CONCLUSIONS

**Figure 2:**
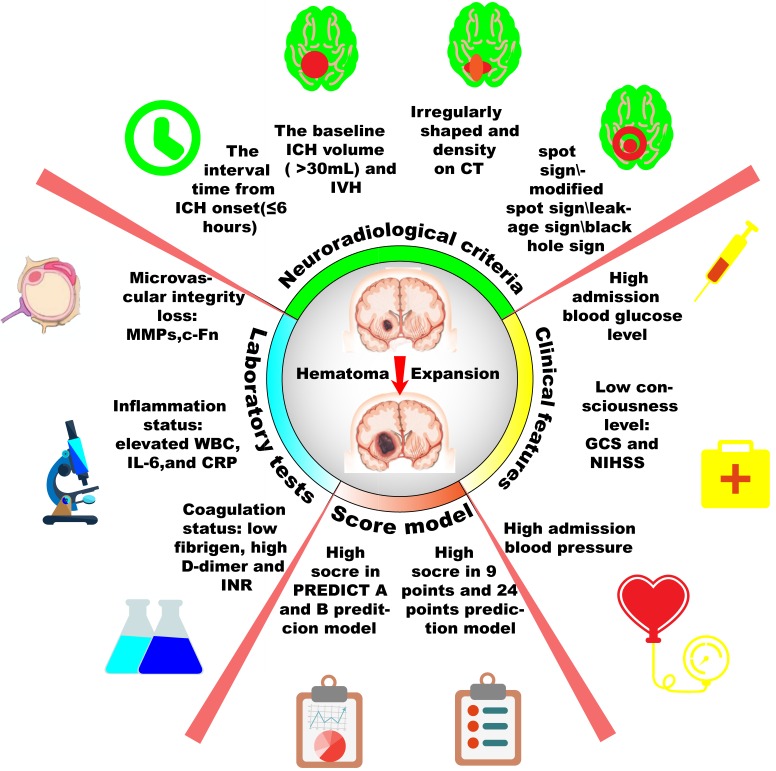
Scheme diagram of predictors for hematoma expansion after intracerebral hemorrhage Hematoma expansion may be predicted by those four aspects: laboratory tests, clinical features, neuroradiological criteria and prediction score model. More accurate prediction may lead better stratification and intensive therapies to patients destined to poor prognosis.

One third of ICH patients would may occur HE, which is strongly predictive of worse prognosis and potentially preventable. Potential HE predictors could help clinicians to better stratify patients, who are destined to undergo HE and tailor intensive therapies timely and effectively. In this review, we discuss the definition and pathophysiology of HE and summarize individual or combined predictors of HE, which include clinical (GCS, NIHSS, blood pressure, blood glucose), laboratory (anticoagulation, inflammation and microvascular integrity), and radiographic (interval time from onset, the baseline ICH volume, the shape and density on CT, IVH, spot sign parameters, modified spot signs) variables, as well as prediction scores (9-point, BRAIN, PREDICT A/B) (Figure 2). This review also discusses those predictors’ practical utility, internal pathogenesis and future research direction. Further elucidation of the pathogenesis and emergence of better predictors are still required to promote novel and aggressive medical therapeutic interventions via predicting and ameliorating HE after symptom onset.
